# Determination of some quality properties of “hamsi kaygana” prepared with different additives

**DOI:** 10.1002/fsn3.578

**Published:** 2018-01-12

**Authors:** Latif Taşkaya, Elif Yaşar

**Affiliations:** ^1^ Fisheries Faculty Department of Fishing and Seafood Processing Technology Muğla Sıtkı Koçman University Muğla Turkey; ^2^ Agromey‐Agromey Gıda Yem San. ve Tic.Ltd.Şti. İzmir Turkey

**Keywords:** *Engraulis encrasicolus*, European anchovy, functional food, hamsi kaygana, shelf life

## Abstract

This study was aimed to determine the changes of the local anchovy meal which is known as hamsi kaygana in Turkey during cold storage at +4°C ± 1°C. Physicochemical (pH, TVB‐N, TMA‐N, and TBA) shelf life analyses were carried out for hamsi kaygana samples. It was confirmed that the pH values obtained from research groups were within the limit values of the literature. No statistically significant difference was observed (*p* > .05) between control, stinging nettle, and rosemary groups that were within all created product groups on 12th day of storage, and the observed difference was only present in cumin group (*p* < .05). Upon examining TVB‐N (Total Volatile Basic Nitrogen) values on 12th day of storage, we saw the lowest TVB‐N value (17.01 ± 0.21 mg/100 g) was at stinging nettle group. However; it was found out that highest TVB‐N value belonged to cumin group with the value of 19.38 ± 0.42 mg/100 g. It was found that 12th day TBA (Thiobarbituric Acid) values of all other groups except control group did not exceed limit values. Among TMA‐N (Trimethylamine Nitrogen) values of all groups on 12th storage period, the lowest value belonged to cumin group samples. While the highest TMA‐N value was found as 14.70 ± 0.30 in control group samples. Therefore, the results showed us that using dried herb and spices in hamsi kaygana production and the storage of the products have an influence on shelf life.

## 1 INTRODUCTION

It is a well‐known fact that the shores of Turkey have a rich potential in terms of fishing. Among the most fished species in our country, which is surrounded by sea on three sides, is the European anchovy (*Engraulis encrasicolus* L.1758); and more than 50% of fish fished at seas of our country consists of European anchovy. We see European anchovy especially as the most common type of fish in Black Sea Region of Turkey. According to official fishing statistics of our country, the total amount of marine fish obtained from fishing is 345.765 tones and 193.492 tones of this amount comes from European anchovy production (TUİK, 2015).

The majority of European anchovy in our country is consumed freshly, and a little proportion is used as fish oil or in fish handling (frozen, canning, marinated, etc.). Due to scarcity of alternative handling methods, the consumption of anchovy is limited with seasons (Karaçam, Kutlu, & Köse, 2002; Tırakoğlu, 2003). Consumers rather prefer healthy, high quality, natural, and fresh fish products. In addition to being freshly consumed, the European anchovy is offered for consumption after being processed with cooling and freezing, drying, marinating, and salinization techniques. It is also used to make by‐product such as fish flour and fish oil. Like many other fish types, European anchovy is recently offered for off‐season consumption thanks to freezing technology. Within this context, European anchovy is very suitable as a ready‐to‐eat cooked food due to the fact that it is an abundant and easy to obtained seafood. Food production should be safe, always high quality, healthy, and cheap. It should last a long time, and stay natural and fresh. It should appeal to palate of people, and traditional flavors should not be lost (Kilcast & Subramaniam, 2000). With the advancements in fish handling methods, obtaining new products and extending the shelf life of existing products and maintaining their quality is of utmost importance. This way, it becomes possible to offer fish that is hunted in certain periods for human consumption all the time (Erdem, Bilgin, & Çağlak, 2005). It was indicated that cat fish meat had normal acidity in terms of physicochemical characteristics. It is also indicated that, while sample fillets have a protein content of 17.35%, they have a high level of lipid content (5.32%) (Chwastowska‐Siwiecka, Skiepko, Pomianowski, & Kondratowicz, 2016). In recent years, large investments have been made in canned food and processing industry and new options have been offered to consumers in our country. Fishery production with canned food, freezing, salting, smoking, and marinade technologies is concentrated in coastal regions, mainly in Marmara, Aegean, and Black Sea Regions of Turkey. These developments contribute to the popularization of fish consumption in our country. It is well‐known that fish and other seafood products have an important place in human nutrition.

Both the raw materials and quality parameters of the product are very important in order to offer to consumers healthy, delicious, and functional food products that have high nutritional value and long shelf life. Hamsi kaygana is one of the food types specific to Easter Black Sea Region. In addition to being delicious and nutritional, it is very easy to prepare. In this regard, it is very suitable as a functional food. As with any other functional foods, the quality of substances used for hamsi kaygana is very important. Also, the factors such as the processing method and process, storage conditions, and presentation type influence the quality of these foods (Angelidis et al., 2006). Therefore; in order to evaluate the hamsi kaygana as a functional food, the quality, and shelf life of this product must be determined. In today's world, the need for ready‐to‐eat food has increased greatly. People turn toward ready‐to‐eat foods that are not only easily prepared, but also pose no risk to health. As a result, new products need to be developed in order to meet this increasing demand.

Anchovy is a very important food due to the composition of crude fat and crude protein it contains. Rich in polyunsaturated fatty acids and phospholipids, anchovy is a quite balanced source of food in terms of crude protein and vitamin content (Guner, Dincer, Alemdag, Colak, & Tufekci, 1998; Kinsella, 1987). It was reported that crude fat content value of anchovy (*E. encrasicolus*) were between 5.30% and 13.60% in the Sea of Marmara of Turkey. They explained that the differences may be due to species or other effects such as season and region (Gökoğlu et al., 1999). It was also reported that higher fat content in the same species provided from the Black Sea (Kaya & Turan, 2010; Kocatepe & Turan, 2012). Different studies made on anchovy reported the crude protein ratio as 16.31% (Kocatepe & Turan, 2012), 19.56% (Ayas, 2006), 16.94%–17.36% (Kaya & Turan, 2010). It is indicated that ~30% of total fatty acid amount in anchovy are docosahexaenoic acid (DHA) and eicosatetraenoic acid (EPA) (Guner et al., 1998; Shahidi & Cadwallader, 1997). The positive effects of DHA and EPA on cardiovascular diseases, premature birth, some skin diseases, some cancer types such as colon cancer and lung cancer, certain diseases that prevent brain functions to be performed such as alzheimer were proven with previous studies (Connor, 2000; Huhges, 1995; Olsen & Secher, 2002; Rafflenbeul, 2001).

Anchovy meals have an importance because in addition to their flavor, they are part of a regional and cultural richness with their different flavor and their positive effects on human health. Time honored anchovy meals that are consumed throughout the year include fried stuffed anchovy, pida with anchovy, rice with anchovy, pastry with anchovy, anchovy meatballs, poached anchovy, fried anchovy, anchovy soup, anchovy casserole, soured anchovy, anchovy stuffings, hamsi kaygana, anchovy dessert, bread with anchovy and hamsi kuşu (Boran & Albayrak, 2002). It is scientifically proven that seafood products are more sensitive against microbial and chemical spoilages than other foods (Colombari et al., 2007; Ünlütürk & Turantaş, 1999). Using natural antimicrobial and antioxidant substances as preservatives in foods instead of chemical substances is important for obtaining healthier and safer products. The results of many studies prove that antimicrobial substances obtained from natural resources such as plants successfully preserve the safety of the food (Aktuğ & Karapınar, 1988; Alzokery & Nakahara, 2003; Malicki, Trziszka, Szpak, & Zródlowska‐Danek, 2010; Oussalah, Caillet, Saucier, & Lacroix, 2006). As the interest toward plants and spices increased in our times, so did the number of scientific studies; and a new trend began toward natural antimicrobial substances rather than chemical substances in especially food industry in terms of antimicrobial substances usage.

The first laboratory study about the use of spices to preserve foods was made by researchers named Hoffman and Evans in 1911. Today, even though the spices added into foods are not in concentrations to show antimicrobial activity, the increased interest toward the use of natural preservers rather than current chemical preservers allowed more studies to be made about the antimicrobial effects of spices. Also, the desire to know whether they have any inhibitor effects on starter cultures, which are used in the production of fermented products, showed the importance of these studies (Con, Ayar, & Gökalp, 1998). Spices are natural herbal substances or their mixtures which are obtained directly or by smashing, drying or grinding parts of various plants such as seed, core, fruit, flower, shell, root, leaf; they are added into foods to give color, flavor, smell, and taste, they are not considered food by themselves and they can be effective even when used too little. They are used to work up an appetite, make the taste, color, smell of the food more pleasant and to make digesting easier. One of their important effects is their antimicrobial characteristic (Altuğ, 2001; Çakmakcı & Çelik, 2004). Corbo et al. (2008) studied the effects of natural antimicrobial substances (cumin, thyme, etc.) by inoculating spoilage microorganisms to fish hamburger. They reported that natural antimicrobial substances are effective against the growth of mesophilic and psychotropic bacteria and that they delayed the decomposition of sensory quality. After applying hydrostatic pressure on traditionally produced fish burgers, it was reported that the shelf life of products could be extended up to 6 weeks if they are stored in refrigerator conditions (4°C ± 1°C) (Malicki et al., 2010).

In addition to being an easy‐to‐prepare product with high nutritional value, hamsi kaygana can also be used as a functional food product. Emanet (2010) studied and evaluated the microbiological quality of regional hamsi kaygana and the effect of dried herb and spices, which are used during its production, on *Stophylococcus aureus*. However, as with any other ready‐to‐eat foods, the quality of substances used for hamsi kaygana is very important. Therefore; in order to evaluate the hamsi kaygana as a functional food, the quality, and shelf life of this product must be determined. The objective of this study is to determine the effects of dried stinging nettle, rosemary, and cumin, which are used as natural preservatives in hamsi kaygana production, on product quality. Consequently, the effects of dried herb and spices on shelf life and quality of cooked hamsi kaygana which is stored in refrigerator conditions (4°C ± 1°C).

## 2 MATERIAL AND METHODS

### 2.1 Production materials

The European anchovies that were used in hamsi kaygana production were bought from a local hypermarket (HORECA; Metro Wholesaler Market‐İzmir), in frozen fillet form, eviscerated, and clean. Moreover, the purchased anchovies that do not exceed the consumption date (expire date) were used. Anchovies, stored under frozen conditions (−18°C), were purchased at a local hypermarket. The frozen anchovies used in the production of hamsi kaygana were approximately 14 kg. The frozen anchovies were thawed in the refrigerator conditions for during 24 hr before making hamsi kaygana. Dried stinging nettle, rosemary, and cumin, which are used as natural preservatives in hamsi kaygana production, was obtained from an herbalist (Arifoglu Baharat Gıda San. Ve Ltd. Şti.). Salt, olive oil, egg, bread, and green onion, which are used in hamsi kaygana production, was bought from Metro Cash & Carry İzmir store. And here is a quick rundown about the stinging nettle, rosemary, and cumin which are used within the scope of this study; the stinging nettle is a herbaceous plant that blossoms between the months of May–August and belongs to *Urticaceae* family. Its trunks are vertical, square‐like, simple or branching from the base. Its leaf is petiolated, oval‐shaped or toothed, its upper part is dark green colored and bright and covered with biting hair. Rosemary is a perennial plant belonging to *Labiatae* family, originates from Mediterranean, always green, appears in bush form and similar to pine leaf, has a length of 2–3 cm, dark green colored with purple flowers. Cumin is an annual plant type belonging to *Umbelliferae* family, blooms white and pinkish flowers between May and June, and has a length of 40–60 cm. Its origin is from East Mediterranean and Middle East (Akgül, 1993).

### 2.2 Hamsi kaygana production

First of all, the frozen European anchovies (*E. encrasicolus*) were dissolved a day ago in 4°C refrigerator conditions. Dissolved fish are sliced into small pieces with the help of a knife. European anchovies (500 g), green onion (200 g), breadcrumbs (100 g), salt (9 g), 1 egg (~9 g), and olive oil (32 ml) were mixed inside a deep cup carefully without pulverizing in a homogeneous way by hand. The obtained homogenous mixture was put inside a microwave oven (White Westinghouse, 1,200 watt) tray (heat‐resistant glass) and cooked for a duration of 16 min. Cooking condition (at 1,200 watts for 16 min) for all experimental group was the same. After the cooked products are cooled in room temperature, they are cut into slices, put into locked refrigerator packages and stored at refrigerator condition (4°C ± 1°C). They were treated as hamsi kaygana control group that will be produced without dried herb and spice preservatives. Test groups consisted of three groups that included stinging nettle, rosemary, and cumin. The anchovies and green onion were chopped down into small pieces. The chopped anchovy (500 g), green onion (200 g), breadcrumbs (100 g), salt (9 g), one egg (~9 g), and olive oil (32 ml) were mixed thoroughly to the dough consistency in a separate mixing bowl for each group. The control group was cooked at 1,200 watts for 16 min in a microwave oven in a heat‐resistant glass container without the addition stinging nettle, rosemary, and cumin. In the other groups, 2 g stinging nettle (0.3%) was added in group stinging nettle, 4 g rosemary (0.5%) in group rosemary, and 4.5 g cumin (0.6%) in group cumin and it was mixed and cooked in the same way as the control group. After the cooked products are cooled in room temperature. The cooled and sliced hamsi kaygana was placed on locked refrigerator packages and stored at refrigerator condition (4°C ± 1°C). For the analyses, random packages were selected on days 1, 4, 6, 8, and 12 and each analysis was performed with three parallels.

### 2.3 Physicochemical analyzes

During pH measurements of test groups; 5 ml deionized water was added on 5 g sample and the sample was torn into pieces with ultra turrax for 1 min. And then, pH of the sample was measured in room temperature with pH meter (Lima Dos Santos, James, & Teutscher, 1981). TVB‐N amount of sample groups was determined with Lücke‐Geidel method modified by Antoacopoulus (Ludorf & Meyer, 1973). TBA (mg malondialdehyde (MDA)/kg) analysis was conducted to determine the lipid oxidation of all test groups. Distillation process was conducted by weighing 10 g of sample, and then homogenizing it with 97.5 ml distilled water and 2.5 ml 4 N HCl. 5 ml was taken from collected distillate and put into tubes. 5 ml. reactive was added on top of it. The sample tubes were kept in 90°C water‐bath for 35 min. After they are cooled, measurement is taken with spectrophotometer (Tarladgis, Watts, & Yonathan, 1960). During the period of storing test groups, Trimethylamine Nitrogen (mg TMA‐N/100 g) analysis was conducted based on AOAC (1984) method.

### 2.4 Statistical analysis

All the experiments were performed at least in triplicate. The results were reported as means ± standard deviation (SD). The statistical evaluation of obtained analysis data was conducted (SPSS, 1999 Version 15.0 Chicago, IL, USA). The data obtained from the research were subjected to variance analysis using SPSS 15.0 for Windows package software and evaluated at (*p* < .05) confidence interval using Duncan's Multiple Test.

## 3 RESULT AND DISCUSSION

### 3.1 Changes in pH values

**Figure 1:**
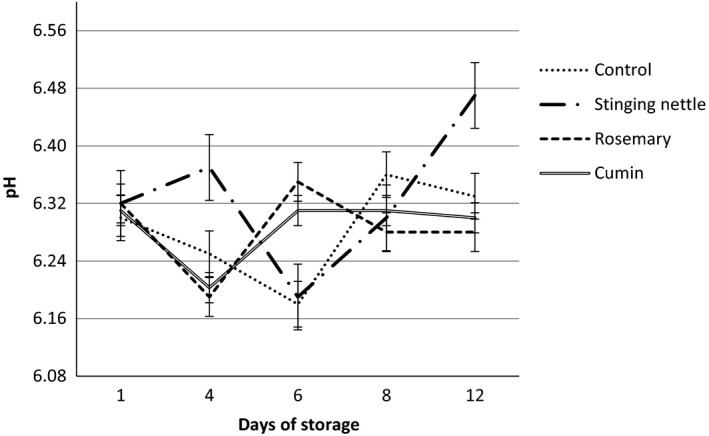
Changes in pH contents of hamsi kaygana samples during cold storage at 4° ± 1°C

The changes that occur in pH values of four groups of hamsi kaygana, namely control group, stinging nettle, rosemary, and cumin, during storage period have been shown in Figure 1. And also; the experiment was carried out in triplicate. In food industry, pH value is one of the important factors that influence microbial and enzymatic activity; determining it and keeping it stable is an important criterion in maintaining product quality (Olgunoğlu, 2007). Known as the measurement of acidity or alkalinity of a product, pH varies from food to food (Court, 2005; McKee, 1995). Banwart (1987) defined food based on their pH value; according to him, foods that have a lower pH value than 3.7 are highly acidic, the ones between 3.70 and 4.60 are acidic, the ones between 4.60 and 5.30 are medium acidic and the ones with higher than 5.3 pH value are food with little or no acid.

As it can be seen in Figure 1, the initial pH values for the produced hamsi kaygana in control, stinging nettle, rosemary, and cumin group were, respectively, measured as 6.30 ± 0.01; 6.32 ± 0.04; 6.32 ± 0.00, and 6.31 ± 0.02. For all product groups that were stored at a temperature of 4°C ± 1°C, no statistically significant difference was observed on 12th storage day between control, stinging nettle, and rosemary groups (*p* > .05), and the observed difference was only present in cumin group (*p* < .05). As a result of the nitrogenous compounds of the test groups and changes due to storage, some changes were observed in pH values. It was found out that pH values obtained from the groups were within limit values of the literature. Ludorf and Meyer (1973) reported that the acceptable upper limit for the pH of fish is 6.8–7.0. It was reported by various researchers that the pH value of seafood meat is an important factor that affects microbial and enzymatic changes (Aksu et al., 1997). They found that the pH values after 12th day for cooked anchovy cakes were between the values of 6.93–7.13 and there was an increase compared to the starting value (İnanlı, Karaton, & Çoban, 2011). It was reported that the pH values of fish meat were between 6.2 and 6.5 after rigor mortis (Suvanich & Marshall, 1998). pH value for fresh fish meat was 6.00–6.50, and it was reported that this value could increase depending on the period of storage and the acceptability value was 6.80–7.00 (Erkan, 2002; Ludorf & Meyer, 1973; Oehlenschlager, 1989; Varlık, Ugur, Gökoglu, & Gün, 1993). A study conducted on anchovy meatballs showed that pH values increased from 6.33 to 6.56 after 10 days of storage (Turhan, Evren, & Yazici, 2001).

### 3.2 Changes in TVB‐N values

**Figure 2:**
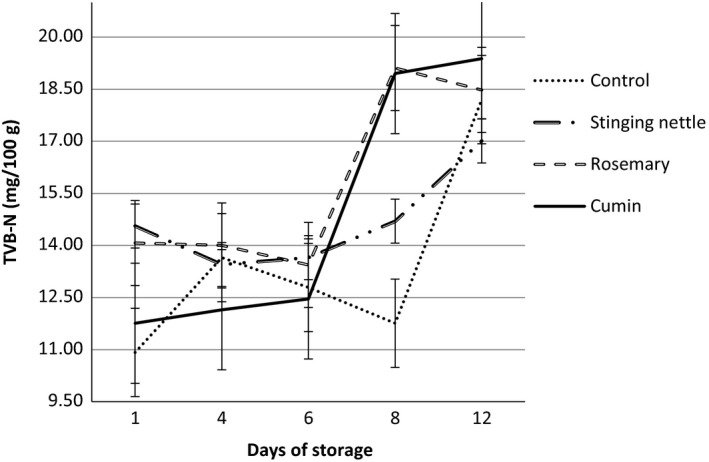
Changes in TVB‐N contents of hamsi kaygana samples during cold storage at 4°C ± 1°C

TVB‐N data are an important criterion in evaluation of chemical degradation and loss of freshness of fish. In this regard, changes in TVB‐N values of all groups throughout storage (4°C ± 1°C) by day are given in Figure 2. Each measurement was performed in triplicate. Quality classification of fish and seafood according to TVB‐N values is defined as below (Varlık et al., 1993). It is stated that it is categorized as “very good” up to 25 mg/100 g TVB‐N; “good” up to 30 mg/100 g TVB‐N; “marketable” up to 35 mg/100 g TVB‐N; and “spoilt” over 35 mg/100 g TVB‐N.

Among all the hamsi kaygana groups produced, it was found that on the 1st day of storage (4°C ± 1°C) the control group had the lowest TVB‐N value of 10.92 ± 0.21 mg/100 g and the stinging nettle group had the highest TVB‐N value (14.56 ± 0.12 mg/100 g). When the TVB‐N values of rosemary and cumin groups on 8th day of storage was analyzed, it was found that there was not any significant difference (*p* > .05) between two groups and that there was a sharp increase in both groups. When the TVB‐N values on the 12th day of storage were analyzed, it was seen that the stinging nettle group had the lowest TVB‐N value (17.01 ± 0.21 mg/100 g). However, it was found that the cumin group had the highest TVB‐N value of 19.38 ± 0.42 mg/100 (Figure 2). Aksu et al. (1997) reported that marinated anchovy had an initial TVB‐N value of 8.3 mg/100 g, but the initial TVB‐N value increased to 15.1 mg/100 g at the end of storage (150 days) period. One of the most common chemical indicators of the degradation of marine fish is known as Total Volatile Base Amines (Zhong‐Yi, Zhong‐Hai, Miao‐Ling, & Xiao‐Ping, 2010) The pomegranate sauce was used to prepare for anchovy marinades. The TVB‐N value was determined as 8.99 mg/100 g in anchovy marinades at the end of the 21‐month storage at 4°C (Gökoğlu, Topuz, & Yerlikaya, 2009). It was also found that TVB‐N values were reported in freshly caught fish was between 5 and 20 mg/100 g (Boran & Köse, 2007). According to the TVB‐N analysis results obtained on the final day of storage, it was seen that all groups remained within acceptable limits. It is considered that the TVB‐N analysis results were obtained depending on the dominance of microbial load on product groups according to the storage of dried herbs and spices which are used in the production of hamsi kaygana. Within this framework, TVB‐N was pointed out as one of the most common tests used for the evaluation of fish quality (Volpe et al., 2015). In a study on the determination of microbiological quality of hamsi kaygana stored at +5°C after it was produced, *Staphylococcus aureus*,* Bacillus cereus*,* Escherichia coli* and *Salmonella* spp. analyses were conducted. Moreover, total mesophilic aerobic bacteria (TMAB) count analyses were also conducted during storage. During storage, number of TMAB continuously increased in all groups and it was found to be approximately 6 log‐kob/g values on the 8th day. *S*. *aureus*,* E*. *coli,* and *Salmonella* spp. analyses were found to be negative in all groups. On the 8th day of storage, *B*. *cereus* counts were found to be approximately 5 log‐kob/g levels in all groups except for hamsi kaygana with cumin. As a final phase, high (6 log‐kob/g) and low (3 log‐kob/g) doses of *S*. *aureus* were inoculated into hamsi kaygana with cumin which produced better results for *B*. *cereus* with comparison to other groups and the samples were stored at +5°C and counted for 6 days long (Emanet, 2010). As a result of 150‐days storage at −18°C of hamsi kuşu which are made of salted and unsalted anchovies, it was found that the TVB‐N value reached 12.72 mg/100 g and 9.37 mg/100 g, respectively (Köse, Karaçam, Kutlu, & Baran, 2001). Some studies on improving the quality of ready‐to‐eat seafood products with the addition of spices are being conducted. They reported that the TVB‐N value of sardine used in marinating technology kept increasing throughout refrigerator conditions (Gökoğlu et al., 1999). It was reported that the increase in TVB‐N was resulted from endogenous enzyme activity and bacterial development (Duman & Özpolat, 2012; Howgate, 2010; Xu et al., 2014).

### 3.3 Changes in TBA values

Biochemical composition of fish meat is important to the consumer as much as it is to the industrialists who use the fish as raw material and process it into various products. White meat fish such as whiting (*Merlangius merlangus*), red mullet (*Mullus barbatus barbatus*), and brill (*Scophthalmus rhombus*) have 1% of lipid per their weight and other fatty fish such as mackerel (*Scomber scombrus*), sardine (*Sardinops sagax*), salmon (*Salmo salar*), anchovy (*Engraulis anchoita*), horse mackerel (*Trachurus japonicus*), tunny fish (*Thunnus thynnus*), and trout (*Salmo trutta*) have a lipid ratio between 5% and 25% per their weight. In general, fresh water fish apart from trout has lesser lipid. Fish are classified into three groups as fatty fish, semifatty fish and nonfatty fish (Huss, 1988). It was stated that the lipid ratio in anchovy (*E. encrasicolus*) is 4.72% (Ayas, 2006), 6.49%–16.32% (Öksüz & Özyılmaz, 2010), 10.04% (İnanlı, Özpolat, Çoban, & Karaton, 2010).

Amount of malondialdehyde in food is generally associated with oxidative rancidity and the rancidity can be measured by the number of TBA (Cai et al., 2014; Koning & Silk, 1963; Sinnhuber & Yu, 1958). Fish is more prone to lipid oxidation with comparison to other meats owing to the highly unsaturated oils in it (Ramanathan & Das, 1992). Fat and fatty acid composition in fish are not steady. They may change depending on the seasonal variations (temperature, salinity, etc.) and also depending on such factors as the lifecycle of fish and fatty acid composition of food that fish eat (Gökoğlu et al., 1999; Ingólfsdóttir, Stefánssson, & Kristbergsson, 1998; Pyz‐Łukasik, Szpetnar, Paszkiewicz, Tatara, & Brodzki, 2016; Zlatanos & Laskaridis, 2007). In the studies conducted until now, it has been pointed out that the TBA contents are good indicators in determination of quality of fish which are frozen, chilled, or stored in ice (Kuusi, Nikkila, & Savolainen, 1975; Tarladgis et al., 1960; Vareltzis, Zetau, & Tsiaras, 1988). It is reported in many studies that there is a correlation in terms of rancidity between TBA values and sensory analysis in fatty fish. It is especially pointed out that it is an important method in determination of lipid oxidation in fish species (Barnett & Nelson, 1991; Ramanathan & Das, 1992; Tarladgis et al., 1960). The lipid in fish decomposes as a result of contact with lipolytic and lipoxidative enzymes and air during processing and storage and, by producing oxidative products, it may become rancid through oxidation which causes high level of rancidity (Kutlu, 1996; Soyer, 1999). Therefore, it was reported that the number of thiobarbituric acid used in determination of rancidity degree of lipid as indicated above (Erdem et al., 2005) should be at least 3 in a very good material and at most 5 in a good material and the limit value for acceptability was between 7 and 8 (Schormüller, 1968; Varlık et al., 1993).

**Figure 3:**
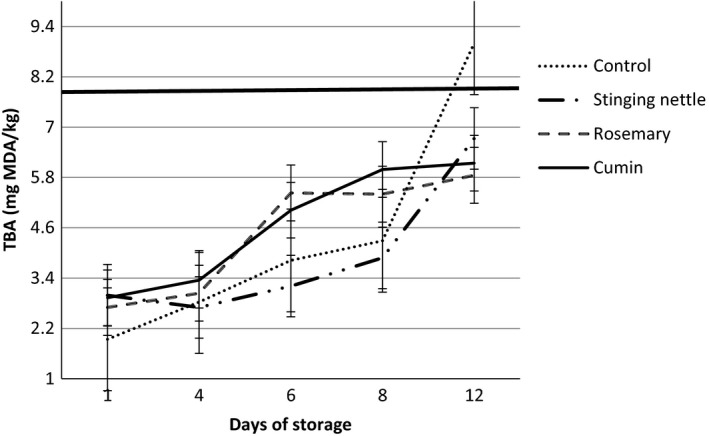
Changes in TBA contents of hamsi kaygana samples during cold storage at 4°C ± 1°C

Changes in TBA values of all groups throughout storage at 4°C ± 1°C by day are given in Figure 3. The experiment was carried out in triplicate. It was found that there was a significant difference between the control group and other groups on the 1st day of storage (*p* < .05). It was seen that the control group had the lowest value of 1.94 ± 0.19 (mg·MDA/kg) on the 1st day. The value which was found for the control group in the first day of storage was found out to be 9.00 ± 0.37 (mg·MDA/kg) on the 12th day of storage. We can see that the TBA value of control group exceeded the threshold value on 12th day. It can be said that all groups had a graduated increase in TBA values depending on the storage period. It was seen that the TBA values of other three groups which are added with dried herbs and spices did not reach acceptability upper limit on the same day of storage. However, it was found that other groups apart from the control group did not exceed the threshold values for TBA on 12th day. It was found that the rosemary group had the lowest TBA value (5.85 ± 0.37 mg·MDA/kg) on the 12th day of storage of all groups. It is reported in many studies that there is a correlation in terms of rancidity between TBA values and sensory analysis in fatty fish species. It is especially pointed out that it is an important method in determination of lipid oxidation in fish species (Barnett & Nelson, 1991; Li, Bland, & Bechtel, 2017; Ramanathan & Das, 1992). As a result of the study, it was found out in the statistical comparison between TBA values of the experimental groups depending on the start and end time of storage that the increase is significant (*p* < .05). It was considered that the presence of O_2_ may cause the increase in TBA values of the prepared products and that further studies should be conducted on the packaging methods of the products.

Fried and nonfried hamsi kuşu which was prepared using salted and unsalted anchovy was stored and it was found out that the TBA value showed an irregular change within that period (Köse et al., 2001). While the good quality upper limit for fish meat which is frozen, chilled, or stored in ice as per their TBA values is accepted to be 5 mg malondialdehyde/kg meat, it was reported that the upper limit for acceptability is 8 mg malondialdehyde/kg meat (Schormüller, 1968). In a study, it was reported that the spices (garlic, onion, clove, cinnamon) used in meatball and kebab production displayed a maximum antibacterial effect against the bacteria which cause spoilage and food poisoning (El‐Khateib, Ahmed, & Makboul, 1989). Similarly, Lee et al. (2005) indicated that the rosemary that they used in their studies on ready‐to‐eat hamburger was effective on the total number of living organisms. Quitral et al. (2009) prepared an ice with pheasant's eye and rosemary extract in a study that they conducted. They stored Chilean jack mackerel in that ice with herb extract at 4°C for 23 days. As a result of the study, it was reported that the ice prepared with pheasant's eye and rosemary extract had a positive effect on chemical quality. Köse et al. (2001) concluded that the fried or nonfried “hamsi kuşu,” when prepared with good quality fresh raw materials, have marketable quality up to 3 months when stored at 18°C ± 1°C. Gökoglu, Cengiz, and Yerlikaya (2004) made a study on the effect of pomegranate sauce on marinated anchovy throughout storage period at 4°C. As a result of the study, they stated that the pomegranate sauce had a high antioxidant feature and could be used in marinating technology.

### 3.4 Changes of TMA‐N values

Trimethylamine (TMA) is a compound which is originated from trimethylamineoxide (TMAO) which is an osmoregulator and is present in the muscles of fish and which is responsible for unpleasant odors which can be sensed when the fish is spoiled to the highest degree. TMA‐N occurs as a result of the decomposition of TMAO into trimethylamine after bacterial and enzymatic activities. Amount of TMA‐N in fish increases in parallel with spoilage and especially acts as an indicator in determining the bacterial spoilage in some fish species. It is reported that TMA‐N limit value should be 10–15 mg/100 g as per international standards, (Schormüller, 1968). Ludorf and Meyer (1973) specified the limit values in fish species as; “good” up to 4 mg/100 g, “marketable” up to 10 mg/100 g, “spoiled” up to 12 mg/100 g. Varlık et al. (1993) pointed out that the TMA‐N value in acceptable seafood should be 1–8 mg/100 g and that the products exceeding the value of 8 mg/100 g are spoiled. On the other hand, Surendran et al. (1989) defined this value as 12 mg/100 g.

**Figure 4:**
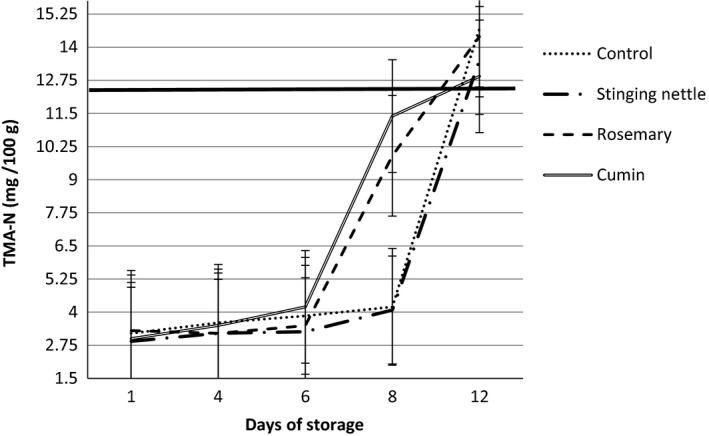
Changes in TMA‐N contents of hamsi kaygana samples during cold storage at 4°C ± 1°C

TMA‐N values of all four groups including the control group and the groups with the addition of dried herb and spices at the end of storage at 4°C ± 1°C are given in Figure 4. And, each measurement was performed in triplicate. Despite the fact that the TMA‐N value of control group at the beginning was measured as 3.20 ± 0.17 (mg/100 g), it was found that this value was approximately five times more (14.70 ± 0.30 mg/100 g) than the value at the beginning on 12th day. It was seen that the value measured exceeded the spoilage value of TMA‐N. Similarly, despite the fact that the TMA‐N value of stinging nettle group at the beginning was measured as 2.90 ± 0.17, it was found that this value was approximately seven times more (13.50 ± 0.30) than the value at the beginning on 12th day. It was seen that the value measured exceeded the spoilage value of TMA‐N. First TMA‐N value of the group containing rosemary was found to be 3.30 ± 0.00 mg/100 g before storage. On the 8th day of storage, a TMA‐N value was found as 9.90 ± 0.30 mg/100 g which is a rapid increase with three times more than the first value. This value could be considered as “marketable” TMA‐N value. However, TMA‐N value on 12th day of storage was found to be not as much as five times more than the first value (14.40 ± 0.00 mg/100 g). First TMA‐N value of the group containing cumin was found to be 3.30 ± 0.0. On the 8th day of storage, a TMA‐N value was found as 11.40 ± 0.60 mg/100 g which is a rapid increase with nearly four times more than the first value. It was seen that the value measured was a value close to spoilage value of TMA‐N (12 mg/100 g). However, TMA‐N value on 12th day of storage was found to be 12.90 ± 0.30. It was found that the cumin group had the lowest value of TMA‐N among all groups on 12th day of storage. On the other hand, the highest TMA‐N value was found in the samples of control group as 14.70 ± 0.30. In a study on anchovy cake which is made using anchovy meat, it was found that while the TMA‐N value of cooked cake was 3.14 mg/100 g at first, it increase to 4.11 mg/100 g at the end of 12th day (İnanlı et al., 2011). In a study on making döner from anchovy and determining the quality specifications of it, TMA‐N value of raw anchovy was found to be 0.84 mg/100 g. It was found that the TMA‐N value after the anchovy döner was made was increase to 5.25 mg/100 g. (İzci et al., 2016). Serdaroğlu (2005) specified that rosemary extract and onion juice, which were used in minced sardine meat that was stored at −20°C for 5 months, had an effect on the delay of oxidation for 3 months. In a study which was made on anchovy cake, it was reported that the 3.14 mg/100 g of TMA‐N value after the cake was cooked reached 4.11 mg/100 g (İnanlı et al., 2011). The studies conducted suggest that the spices used are not only effective in adding a flavor or taste, but they are also important for the preservative effect in food conservation (McLay, 1972; Nair, Vasudevan, & Venkitanarayanan, 2005).

## 4 CONCLUSION

The products which are to be used as human food should not contain any risk which threatens public health. Effects of dried herbs and spices which are used in the production of hamsi kaygana on the storage of products were examined. It is considered that the TVB‐N analysis results on the experimental groups of additives were obtained depending on the limitation of microbial loads of the products. TVB‐N value on 12th day indicated that the stinging nettle was much more effective than the cumin when used in hamsi kaygana production. At the end of the storage, the highest TVB‐N value was obtained in the cumin group with 19.38 ± 0.42 mg/100 g. However, until the end of the storage day, the consumable limit values were not exceeded in any group. It was seen that there were changes in product pH resulting from the spoilage of compounds with nitrogen contained in the product groups. At the end of the storage period, the pH value was determined to be 6.33 ± 0.01, 6.47 ± 0.02, 6.28 ± 0.01, and 6.30 ± 0.00 for groups control, stinging nettle, rosemary, and cumin, respectively. The highest value (6.47 ± 0.02) at the end of the storage was obtained in group stinging nettle. Whereas it was found that the rosemary group had the lowest value (6.28 ± 0.01) of the pH value among all groups on 12th day of storage. TBA is one of the parameters which are used in determination of shelf life of the prepared products with regard to storage. The TBA values increased in all groups during storage period. It was found that the rosemary group had the lowest value (5.85 ± 0.37 mg·MDA/kg) of TBA among all groups on 12th day of storage. On the other hand, the highest TBA value (9.00 ± 0.37 mg·MDA/kg) was found in the samples of control group. It was found that the rosemary (5.85 ± 0.37 mg·MDA/kg) used in the production is much more effective than the stinging nettle (6.73 ± 0.08 mg·MDA/kg) and cumin (6.14 ± 0.18 mg·MDA/kg) in terms of product shelf life. The TMA‐N values increased in all groups and the highest increase was observed in the control group (14.70 ± 0.30 mg/100 g) on the 12th day of storage. The lowest TMA‐N value (12.90 ± 0.30 mg/100 g) was obtained in the cumin group at the end of the storage day. It was found that the control group had the highest value (14.70 ± 0.30 mg/100 g) of TMA‐N among all groups on 12th day of storage of the produced hamsi kaygana. On the other hand, the lowest TMA‐N value (12.90 ± 0.30 mg/100 g) was found in the cumin group. It was found that the cumin used in the production is much more effective than the stinging nettle (13.50 ± 0.30 mg/100 g) and rosemary (14.40 ± 0.00 mg/100 g) in terms of product shelf life. Moreover, in addition to obtained data, it is foreseen that further studies should be conducted on using different packaging methods for the prepared experimental groups. Furthermore, various studies on the amount of dried herbs and spices used in the experiments are needed to be conducted. As a result of the study conducted, the importance of the alternative consumable food product, which is obtained from anchovy which has a high potential of consumption in our country, pointed out. Moreover, it is considered that hamsi kaygana will make positive contribution in increasing the annual consumption rates of seafood. It is predicted that hamsi kaygana and its possibility of becoming widespread may make contribution in processed seafood range.

## CONFLICT OF INTEREST

The author declare no conflicts of interest.

## References

[fsn3578-bib-0001] Akgül, A. (1993). Baharat bilimi ve teknolojisi. Gida Teknolojisi Dernegi Yayinlari, 15, 77–79.

[fsn3578-bib-0002] Aksu, H. , Erkan, N. , Çolak, H. , Varlik, C. , Gökoğlu, N. , & Uğur, M. (1997). Farkli asit tuz konsantrasyonlariyla hamsi marinati üretimi esnasinda olusan bazi degisiklikler ve raf omrunun belirlenmesi. Yuzuncuyil Universitesi Veteriner ve Hayvancilik Dergisi, 8(1), 83–87.

[fsn3578-bib-0003] Aktuğ, S. E. , & Karapınar, M. (1988). Sensitivity of some common food poisoning bacteria to thyme, mint and bay leaves. International Journal of Food Microbiology, 3(6), 349–354.

[fsn3578-bib-0004] Altuğ, T . (2001). Gida katki maddeleri (pp. 128–130). Izmir‐Turkey: Ege Universitesi Basimevi.

[fsn3578-bib-0005] Alzokery, N. S. , & Nakahara, K. (2003). Antibacterial activity of extracts from some edible plants commonly consumen in Asia. International Journal of Food Microbiology, 80, 223–230. 12423924 10.1016/s0168-1605(02)00169-1

[fsn3578-bib-0006] Angelidis, A. S. , Chronis, E. N. , Papageorgiou, D. K. , Kazakis, I. I. , Arsenoglou, K. C. , & Stathopoulos, G. A. (2006). Non‐lactic acid contaminating flora in ready‐to‐eat foods: A potential Food‐Quality Index. Food Microbiology, 23, 95–100. https://doi.org/10.1016/j.fm.2005.01.015 16942992 10.1016/j.fm.2005.01.015

[fsn3578-bib-0007] AOAC . (1984). Official methods of analyses. (14th ed). Washington, DC, USA: Association of Official Analytical Chemists.

[fsn3578-bib-0008] Ayas, D. (2006). Gokkusagi alabaligi (*Oncorhyncus mykiss*), hamsi (*Engraulis encrasicolus*) ve sardalya (*Sardina pilchardus*)’nin sicak tutsulenmesi sonrasindaki kimyasal kompozisyon oranlarindaki degisimleri. EU Journal of Fisheries & Aquatic Sciences Cilt, 23, 343–346.

[fsn3578-bib-0009] Banwart, G. J . (1987). Basic food microbiology (2nd edn) (pp. 749). USA: Department of microbiology, The Ohio State University.

[fsn3578-bib-0010] Barnett, H. J. , & Nelson, W . (1991). A comparative study using multipleindices to measure changes in quality of pink and coho salmon during fresh and frozen storage. NOAA Technical Memerandum NMFS F/NWC.

[fsn3578-bib-0011] İzci, L. , Bilgin, Ş. , Günlü, A. , Çetinkaya, S. , Diler, A. , Genç, Yİ. , & Bolat, Y. (2016). Hamsi baligi (*Engraulis encrasicolus*) donerinin soguk depolama sirasindaki kalite degişimleri. Journal of Agricultural Sciences, 22, 360–369.

[fsn3578-bib-0012] Boran, G. , & Albayrak, N . (2002). Karadeniz bolgesi’ nin yoresel hamsi yemekleri ve hamsinin besin miktarindaki mevsimsel degisim. Yuzuncu Yil Universitesi, Gida Muhendisligi Bolumu Dergisi 1–6.

[fsn3578-bib-0013] Boran, M. , & Köse, S. (2007). Storage properties of three types of fried whiting balls at refrigerated temperatures. Turkish journal of Fisheries and Aquatic Sciences, 7, 65–70.

[fsn3578-bib-0014] Cai, L. , Wu, X. , Dong, Z. , Li, X. , Yi, S. , & Li, J. (2014). Physicochemical responses and quality changes of red sea bream (*Pagrosomus major*) to gum arabic coating enriched with ergothioneine treatment during refrigerated storage. Food Chemistry, 160, 82–89. https://doi.org/10.1016/j.foodchem.2014.03.093 24799212 10.1016/j.foodchem.2014.03.093

[fsn3578-bib-0015] Çakmakcı, S. , & Çelik, U . (2004). Gida katki maddeleri pp (201–214). Erzurum‐Turkey: Ataturk Universitesi.

[fsn3578-bib-0016] Chwastowska‐Siwiecka, I. , Skiepko, N. , Pomianowski, J. F. , & Kondratowicz, J. (2016). Pomiary morfometryczne i ocena jakości mięsa suma afrykańskiego. Medycyna Weterynaryjna, 72(2), 102–109.

[fsn3578-bib-0017] Colombari, V. , Mayer, M. D. , Laicini, Z. M. , Mamizuka, E. , Franco, B. D. , & Destro, M. T. (2007). Foodborne outbreak caused by *Staphylococcus aureus*: Phenotypic and genotypic characterization of strains of food and human sources. Journal of Food Protection, 70, 489–493. https://doi.org/10.4315/0362-028X-70.2.489 17340888 10.4315/0362-028x-70.2.489

[fsn3578-bib-0018] Con, A. H. , Ayar, A. , & Gökalp, H. Y. (1998). Bazi baharat ucucu yaglarinin cesitli bakterilere karsı antimikrobiyal etkisi. Gıda, 23(3), 171–175.

[fsn3578-bib-0019] Connor, W. E. (2000). Importance of n‐3 fatty acids in health and disease. American Journal of Clinical Nutrition, 71, 171–175. 10.1093/ajcn/71.1.171S 10617967

[fsn3578-bib-0020] Corbo, M. R. , Speranza, B. , Filippone, A. , Granatiero, S. , Conte, A. , Sinigaglia, M. , & Del Nobile, M. A. (2008). Study on the synergic effect of natural compounds on the microbial quality decay of packed fish hamburger. International Journal of Food Microbiology, 127, 261–267. https://doi.org/10.1016/j.ijfoodmicro.2008.07.014 18804302 10.1016/j.ijfoodmicro.2008.07.014

[fsn3578-bib-0021] Court, A . (2005). Determination of product shelf life food safety authority of Ireland. Guidance note, 18.

[fsn3578-bib-0022] Duman, M. , & Özpolat, E. (2012). Chemical and sensory quality changes of different formulated inegöl fish balls, made from capoeta trutta (Heckel, 1843) during froze storage (‐18 ± 2 C). Gıda, 37, 25–31.

[fsn3578-bib-0023] El‐Khateib, T. , Ahmed, S. H. , & Makboul, M. A. (1989). Trials for increasing keeping quality of egyptian minced meat “Koefte” and “Kaebap” by spice extracts. Proceedings International Congress of Meat Science and Technology, 35(2), 486–497.

[fsn3578-bib-0024] Emanet, G. (2010). Yoresel hamsi kaygana’ nin mikrobiyolojik kalitesinin saptanması ve uretiminde kullanilan kurutulmus ot ve baharatlarin Staphylococcus aureus uzerine etkisi. İzmir‐Turkey: Ege Universitesi Fen Bilimleri Enstitüsü.

[fsn3578-bib-0025] Erdem, M. E. , Bilgin, S. , & Çağlak, E. (2005). Tuzlama ve marinasyon yontemleri ile islenmis istavrit baliginin (*Trachurus mediterraneus*) muhafazasi sirasindaki kalite degişimleri. Ondokuz Mayıs Üniversitesi Eğitim Fakültesi Dergisi, 20(3), 1–16.

[fsn3578-bib-0026] Erkan, N . (2002). Sogukta depolanan bazi balik cinslerinde kullanilan koruyucu katki maddelerinin raf omrune etkisi. Istanbul Universitesi Fen Bilimleri Enstitusu Su Urunleri Avlama ve Isleme Teknolojisi A.B.D. Isleme Teknolojisi Bolumu, Istanbul‐Turkey.

[fsn3578-bib-0028] Gökoglu, N. , Cengiz, E. , & Yerlikaya, P. (2004). Determination of shelf life of marinated sardine (Sardina pilchardus) stored at 4 C. Food Control, 15, 1–4. https://doi.org/10.1016/S0956-7135(02)00149-4

[fsn3578-bib-0029] Gökoğlu, N. , Özden, Ö. , Erkan, N. , Taçnur, T. , Metin, B. , & Metin, S. (1999). Seasonal variation in fat content of anchovy (*Engraulis encrasicolus*). International Journal of Food Science and Technology, 34, 401–402. https://doi.org/10.1046/j.1365-2621.1999.00285.x

[fsn3578-bib-0030] Gökoğlu, N. , Topuz, O. K. , & Yerlikaya, P . (2009). Effects of pomegranate sauce on quality of marinated anchovy during refrigerated storage. Lebensmittel‐Wissenschaft & Technologie, 42(1), 113–118. http://dx.doi.org/10.1016/j.lwt.2008.04.007.

[fsn3578-bib-0031] Guner, S. , Dincer, B. , Alemdag, N. , Colak, A. , & Tufekci, M. (1998). Proximate composition and selected mineral content of commercially important fish species from the Black Sea. Journal of the Science of Food and Agriculture, 78, 337–342. https://doi.org/10.1002/(ISSN)1097-0010

[fsn3578-bib-0032] Howgate, P. (2010). A critical review of total volatile bases and trimethylamine as indices of freshness of fish. Part 2. Formation of the bases, and application in quality assurance. Electronic Journal of Environmental, Agricultural and Food Chemistry, 9, 58–88.

[fsn3578-bib-0033] Huhges, D. A. (1995). Fish oil and the immune system. Nutrition and Food Science, 95(2), 12–16. https://doi.org/10.1108/00346659510078240

[fsn3578-bib-0034] Huss, H. H . (1988). Fresh fish quality and quality changes (pp. 179–202). Rome‐Italy: FAO.

[fsn3578-bib-0035] Ingólfsdóttir, S. , Stefánssson, G. , & Kristbergsson, K. (1998). Seasonal variations in physicochemical and textural properties of north Atlantic cod (*Gadus morhua*) mince. Journal of Aquatic Food Product Technology, 7(3), 39–61. https://doi.org/10.1300/J030v07n03_04

[fsn3578-bib-0036] Karaçam, H. , Kutlu, S. , & Köse, S. (2002). Effect of salt concentrations and temperature on the quality and shelf life of brined anchovies. International Journal of Food Science and Technology, 37, 19–28. https://doi.org/10.1046/j.1365-2621.2002.00526.x

[fsn3578-bib-0037] İnanlı, A. G. , Karaton, N. , & Çoban, Ö. E. (2011). Sensorial, chemical and microbiological quality of anchovy cake. African Journal of Biotechnology, 10(48), 9870–9874.

[fsn3578-bib-0038] Kaya, Y. , & Turan, H. (2010). Comparison of protein, lipid, and fatty acids composition of anchovy (*Engraulis encrasicolus*) during the commercial catching season. Journal of Muscle Foods, 21, 474–483. https://doi.org/10.1111/(ISSN)1745-4573

[fsn3578-bib-0039] Kilcast, D. , & Subramaniam, P . (2000). Leather food research association In Kilcast D. & Subramaniam P. (Eds.), The stability and shelf life of food (pp. 1–19). FL, USA: Cambridge England Woodhead Publishing Limited and CRC Press LLC.

[fsn3578-bib-0040] Kinsella J. E. (Ed.) (1987). Sea foods and fish oils in human health and disease. New York‐USA: Marcel Dekker Inc..

[fsn3578-bib-0041] Kocatepe, D. , & Turan, H. (2012). Proximate and fatty acid composition of some commercially important fish species from the Sinop Region of the Black Sea. Lipids, 47, 635–641. https://doi.org/10.1007/s11745-012-3658-1 22322400 10.1007/s11745-012-3658-1

[fsn3578-bib-0042] Koning, A. M. , & Silk, M. H. (1963). The 2‐thiobarbituric acid reagent for determination of oxidative rancidity in fish oils. Journal of the American Oil Chemists Society, 40, 167. 10.1007/BF02632572 5896323

[fsn3578-bib-0043] Köse, S. , Karaçam, H. , Kutlu, S. , & Baran, M. (2001). Investigating the shelf life of the anchovy dish called ‘Hamsi Kuşu’ in frozen storage at ‐18 ± 1 C. Turkish Journal of Veterinary and Animal Sciences, 25, 651–656.

[fsn3578-bib-0044] Kutlu, S. (1996). Salamura hamsilerde dayanma sureleri ve kalite degisimleri. Trabzon‐Turkey: Karadeniz Teknik Universitesi Fen Bilimleri Enstitusu.

[fsn3578-bib-0045] Kuusi, T. , Nikkila, O.E. , & Savolainen, K. (1975). Formation of malonaldehyde in frozen Baltic Herring and its influence on the changes in proteins. Zeitschrift für Lebensmittel‐Untersuchung und ‐Forschung. A, Food research and Technology, 159, 285–289. https://doi.org/10.1007/BF01139581 10.1007/BF01139581 1224801

[fsn3578-bib-0046] Lee, J. W. , Park, K. S. , Kim, T. G. , Oh, S. H. , Lee, Y. S. , Kim, J. H. , & Byun, M. W. (2005). Combined effects of gamma irradiation and rosemary extract on the shelf life of a ready‐to‐eat hamburger steak. Radiation Physics and Chemistry, 72, 49–56. https://doi.org/10.1016/j.radphyschem.2004.01.003

[fsn3578-bib-0047] Li, C. H. , Bland, J. M. , & Bechtel, P. J. (2017). Effect of precooking and polyphosphate treatment on the quality of microwave cooked catfish fillets. Food Sciences and Nutrition, 5, 812–819. https://doi.org/10.1002/fsn3.465 10.1002/fsn3.465 PMC5448386 28572972

[fsn3578-bib-0048] Lima Dos Santos, C. , James, D. , & Teutscher, F . (1981). Guidelines for chilled fish storage experiments (pp. 180–210). Italy: FAO.

[fsn3578-bib-0049] Ludorf W., & Meyer V. (Eds.) (1973). Fische und fischerzeugnisse. Hamburg‐Germany: Paul parey.

[fsn3578-bib-0050] Malicki, A. , Trziszka, T. , Szpak, M. , & Zródlowska‐Danek, J. (2010). Badania nad zastosowaniem lizozymu i octanu sodu w celu przedluzenia trwalosci miêsa drobiowego. Medycyna Weterynaryjna, 66(10), 699–701.

[fsn3578-bib-0051] McKee, L. H. (1995). Microbial contamination of spices and herbs: A review. Lebensmittel‐Wissenschaft & Technologie, 28, 1–11. https://doi.org/10.1016/S0023-6438(95)80004-2

[fsn3578-bib-0052] McLay, R . (1972). Marinades. Ministry of Agriculture, Fisheries and Food. Torry Advisory Note, 56, 10.

[fsn3578-bib-0053] Nair, M. K. M. , Vasudevan, P. , & Venkitanarayanan, K. (2005). Antibacterial effect of black seed oil on *Listeria monocytogenes* . Food Control, 16, 395–398. https://doi.org/10.1016/j.foodcont.2004.04.006

[fsn3578-bib-0054] Oehlenschlager, J. (1989). Die gehalte an flüchtigen aminen und trimethylaminoxid in fangfrischen rotbarchen aus vershiedenen fanggebieten des nnordatlantiks. Archiv für Lebensmittelhygiene, 40, 58.

[fsn3578-bib-0055] Öksüz, A. , & Özyılmaz, A. (2010). Changes in fatty acid compositions of Black Sea Anchovy (*Engraulis encrasicolus* L.1758) during catching season. Turkish Journal of Fisheries and Aquatic Science, 10, 381–385.

[fsn3578-bib-0056] Olgunoğlu, A. A. (2007). Marine edilmis hamside (Engraulis engrasicholus, 1758) duyusal, kimyasal ve mikrobiyolojik degisimler. Fen Bilimleri Enstitusu: Cukurova Universitesi, Adana‐Turkey.

[fsn3578-bib-0057] Olsen, S. F. , & Secher, N. J. (2002). Low consumption of seafood in early pregnancy as a risk factor for preterm delivery: Prospective cohort study. British Medical Journal, 324(23), 1–5. 11859044 10.1136/bmj.324.7335.447 PMC65663

[fsn3578-bib-0058] Oussalah, M. , Caillet, S. , Saucier, L. , & Lacroix, M. (2006). Antimicrobial effects of selected plant essential oils on the growth of pseudomonas putida strain isolated from meat. Meat Science, 73, 236–244. https://doi.org/10.1016/j.meatsci.2005.11.019 22062294 10.1016/j.meatsci.2005.11.019

[fsn3578-bib-0059] İnanlı, A.G , Özpolat, E. , Çoban, Ö.E. , & Karaton, N. (2010). Marine edilmis hamsi baliginin (*Engraulis encrasicolus*, L., 1758) kimyasal bilesimi ve farkli soslarda duyusal degerlendirilmesi. Journal of FisheriesSciences, 4(4), 455–461.

[fsn3578-bib-0060] Pyz‐Łukasik, R. , Szpetnar, M. , Paszkiewicz, W. , Tatara, M. R. , & Brodzki, A. (2016). Free amino acid content in muscle tissue of bighead carp and wells catfish. Medycyna Wet., 72(10), 632–636. https://doi.org/10.21521/mw.5571

[fsn3578-bib-0061] Quitral, V. , Donoso, M. L. , Ortiz, J. , Herrera, M. V. , Araya, H. , & Auborg, S. P. (2009). Chemical changes during the chilled storage of chilean jack mackerel (*Trachurus murphyi*): Effect of a plant‐extract icing system. Food Science and Technology, 10, 10–16.

[fsn3578-bib-0062] Rafflenbeul, W. (2001). Fish for a healthy heart European. Journal of Lipid Science and Technology, 103, 315–317. https://doi.org/10.1002/(ISSN)1438-9312

[fsn3578-bib-0063] Ramanathan, L. , & Das, N. P. (1992). Studies on the control of lipid oxidation in ground fish by some polyphenolic naturel products. Journal of Agriculture and Food Chemistry, 40, 17–21. https://doi.org/10.1021/jf00013a004

[fsn3578-bib-0064] Schormüller, J. (1968). Fette und lipoide (lipids) In Schormüller J. (Ed.), Handbuch der lebensmittel chemie, Band II/2 teil (pp. 872–878). Heidelberg, New York: Springer Verlag Berlin.

[fsn3578-bib-0065] Serdaroğlu, M. (2005). Effects of using rosemary extract and onion juice on oxidative stability of sardine (*Sardina pilchardus*) mince. Journal of Food Quality, 28(2), 109–120. https://doi.org/10.1111/j.1745-4557.2005.00016.x

[fsn3578-bib-0066] Shahidi, F. , & Cadwallader, K. R . (1997). Flavor and lipid chemistry of seafoods (pp. 20–30). Washington DC, USA: American Chemical Society https://doi.org/10.1021/symposium

[fsn3578-bib-0067] Sinnhuber, R. O. , & Yu, T. C. (1958). Characterization of red pigment the thiobarbituric acid determination of oxidative rancidity. Food Research, 23, 626 https://doi.org/10.1111/j.1365-2621.1958.tb17614.x

[fsn3578-bib-0068] Soyer, A. (1999). Balikta lipid oksidasyonunda rol oynayan hucresel faktorler. Gida Teknolojisi Dernegi Yayinlari, 4, 2.

[fsn3578-bib-0069] Surendran, P. K. , Joseph, J. , Shenoy, A. V. , Perigreen, P. A. , Mahadevayer, K. , & Gopakumar, K. (1989). Studies on spoilage of commercially important tropical fishes under iced storage. Fisheries Research, 7(1–2), 1–9. https://doi.org/10.1016/0165-7836(89)90002-7

[fsn3578-bib-0070] Suvanich, V. , & Marshall, D. L. (1998). Influence of storage time and temperature on quality of catfish (*Ictalurus punctatus*) frames. Journal of Aquatic Food Product Technology, 7(1), 61–76. https://doi.org/10.1300/J030v07n01_05

[fsn3578-bib-0071] Tarladgis, B. , Watts, B. M. , & Yonathan, M. (1960). Distilation method for the determination of malonaldehyde in rancidity food. J American Oil Che. Soc., 37(1), 44–48. https://doi.org/10.1007/BF02630824

[fsn3578-bib-0072] Tırakoğlu, T. (2003). Farkli yontemlerle depolanan ve marinat hamsi uretiminde kullanilan hamsinin tazeliginin urunun mikrobiyolojik ve organoleptik kalitesi uzerine etkilerinin saptanmasi. Sağlık Bilimleri Enstitusu Besin Hijyeni ve Teknolojisi Anabilim Dalı. T.C: Uludag Universitesi, Bursa‐Turkey.

[fsn3578-bib-0073] TUİK . (2015). Turkiye istatistik kurumu, su urunleri istatistikleri. T.C. basbakanlik devlet istatistik enstitusu, Ankara‐Turkey.

[fsn3578-bib-0074] Turhan, S. , Evren, M. , & Yazici, F. (2001). Shelf life of refrigerated raw anchovy (*Engraulis encrasicholus*) patties. E. U. J. Fisheries Aquat. Sci. (3‐4), 18(3–4), 391–398.

[fsn3578-bib-0075] Ünlütürk, A. , & Turantaş, F . (1999). Gida Mikrobiyolojisi (İkinci Baski) (pp. 450–485). İzmir‐Turkey: Ege Universitesi Mengi Tan Basimevi.

[fsn3578-bib-0076] Vareltzis, K. , Zetau, F. , & Tsiaras, U. (1988). Textural deterioration of chub mackerel (*Scomber japonicus collias*) and smooth hound (*Mustelus mustelus L*.) in frozen storage in relation to chemical parameters. Lebensmittel‐Wissenschaft & Technologie, 21, 206–211.

[fsn3578-bib-0077] Varlık, C. , Ugur, M. , Gökoglu, N. , & Gün, H. (1993). Su urunlerinde kalite kontrol ilke ve yontemleri. Gida Teknolojisi Dernegi Yayin, 17, 174.

[fsn3578-bib-0078] Volpe, M. G. , Siano, F. , Paolucci, M. , Sacco, A. , Sorrentino, A. , & Malinconico, M. (2015). Active edible coating effectiveness in shelf life enhancement of trout (*Oncorhynchus mykiss*) fillets. LWT‐Food Science and Technology, 60, 615–622. https://doi.org/10.1016/j.lwt.2014.08.048

[fsn3578-bib-0079] Xu, G. , Xue, T. , Shihan, T. , Huabin, Y. , Huawei, S. , & Ruobo, G. (2014). Combined effect of electrolyzed oxidizing water and chitosan on the microbiological, physicochemical, and sensory attributes of american shad (*Alosa sapidissima*) during refrigerated storage. Food Control, 46, 397–402. https://doi.org/10.1016/j.foodcont.2014.06.010

[fsn3578-bib-0080] Zhong‐Yi, L. , Zhong‐Hai, L. , Miao‐Ling, Z. , & Xiao‐Ping, D. (2010). Effect of fermentation with mixed starter cultures on biogenic amines in bighead carp surimi. International Journal of Food Science and Technology, 45, 930–936. https://doi.org/10.1111/j.1365-2621.2010.02215.x

[fsn3578-bib-0081] Zlatanos, S. , & Laskaridis, K. (2007). Seasonal variation in the fatty acid composition of three mediterranean fish–sardine (*Sardina pilchardus*), anchovy (*Engraulis engrasicholus*) and picarel (*Spicara smaris*). Food Chemistry, 103(3), 725–728. https://doi.org/10.1016/j.foodchem.2006.09.013

